# Investigation of Atrazine Sorption to Biochar With Titration Calorimetry and Flow-Through Analysis: Implications for Design of Pollution-Control Structures

**DOI:** 10.3389/fchem.2018.00307

**Published:** 2018-07-30

**Authors:** Chad J. Penn, Javier M. Gonzalez, Isis Chagas

**Affiliations:** ^1^USDA-Agricultural Research Service National Soil Erosion Laboratory, West Lafayette, IN, United States; ^2^Department of Agricultural and Biological Engineering, Purdue University, West Lafayette, IN, United States

**Keywords:** atrazine, biochar, isothermal titration calorimetry, pollution-control, flow-through

## Abstract

Atrazine is one of the most common broad-leaf herbicides used in the world. However, due to extensive use for many years, atrazine often appears in surface and groundwater. Atrazine transport is inhibited by degradation or sorption to soil components, especially organic matter. Biochar is a charcoal-like material produced from pyrolysis of biomass. Due to the amount and type of functional groups found on biochar, this product has shown potential for sorption of atrazine from solution. There is an interest in developing best management practices utilizing biochar to filter atrazine from non-point drainage with pollution-control structures such as blind inlets. The objective of this study was to explore the kinetics and thermodynamics of atrazine sorption to biochar using two different approaches: flow-through sorption cells and isothermal titration calorimetry (ITC). Twenty-five milligrams of an oak (*Quercus* spp.)-derived biochar was suspended in water and titrated 25 times (0.01 mL per titration) with atrazine at three different concentrations, and by a single titration (0.25 mL), with heat of reaction directly measured with ITC. A benchtop atrazine sorption study that simulated the titration experiment was also conducted. A continuous flow-through system was used to quantify the impact of contact time on atrazine sorption to biochar. Atrazine sorption to biochar displayed both exothermic and endothermic signals within each titration, although the net reaction was exothermic and proportional to the degree of sorption. Net enthalpy was −4,231 ± 130 kJ mole^−1^ atrazine sorbed. The existence of both exotherms and endotherms within a single titration, plus observation of an initial fast reaction phase from 0 to 300 s followed by a slower phase, suggested multiple sorption mechanisms to biochar. Results of flow-through tests supported kinetics observations, with the 300 s contact time removing much more atrazine compared to 45 s, while 600 s improved little compared to 300 s. Based on flow-through results, annual atrazine removal goal of 50%, and typical Midwestern U.S. tile drainage conditions, a pollution-control structure implementing this biochar sample would require 32 and 4 Mg for a design utilizing a contact time of 45 and 300 s, respectively. Future work is necessary for estimating degradation of atrazine sorbed to biochar.

## Introduction

Atrazine [6-chloro-*N*-(1-methylethyl)-1,3,5-triazine-2,4-diamine] is an herbicide used to control annual weeds, mostly in corn (*Zea mays*) production. Regarding active ingredient, only glyphosate (N-[phosphonomethyl]-glycine) is applied more in the United States than atrazine, with 32 million kg applied annually (Mueller et al., 2017). While most of the applied atrazine remains in the soil, a portion of it can be transported offsite through runoff and leaching with minimal volatilization. In one of the earliest studies, Hall et al. (1972) applied 0–9 kg ha^−1^ of atrazine to pre-emerged corn and found that average runoff loss of atrazine was 2.4% of the total amount applied. Numerous studies have demonstrated and quantified the loss of atrazine through both leaching to tile drains and surface runoff (Triplett et al., 1978; Glenn and Angle, 1987; Hall et al., 1989; Buhler et al., 1993; Logan et al., 1994; Fermanich et al., 1996; Moore et al., 2001). These losses constitute a non-point source transport of atrazine, which has resulted in hazardous concentrations in drinking water, and groundwater (Thurman et al., 1991; Jayachandran et al., 1994; Biradar and Rayburn, 1995) and surface water ecosystems (Cooper, 1993; Graymore et al., 2001) throughout the world. For example, in a nationwide study from 2002 to 2011, Stone et al. (2014) found atrazine concentrations that exceeded aquatic life benchmarks in ~5% of the U.S. agricultural streams. Elevated concentrations of atrazine in wells and drinking water present an immediate concern for human health (Van Leeuwen et al., 1999; Benotti et al., 2008; Yu et al., 2018).

Atrazine sorbs to both mineral and organic soil components through a variety of mechanisms, although atrazine has the greatest affinity for soil organic matter (Brouwer et al., 1990; Laird and Koskinen, 2008). For example, Laird et al. (1994) found that 68% of atrazine sorption occurred on soil organic matter that accounted for only 11% of the clay weight. Because of its strong interaction with organic matter, organic carbon distribution coefficients (i.e., K_oc_ = K_d_/OC, where OC is the percentage of organic carbon in the sample and Kd is a distribution coefficient that quantifies the amount of sorbent in the solution and sorbed phase) are often reported for comparing the ability of different soils to sorb atrazine (Ma and Selim, 1996). The interaction between atrazine and organic carbon occurs mostly on amine, hydroxyl, carbonyl, amide, and carboxylic acid functional groups (Wang et al., 1992; Weber, 1993; Welhouse and Bleam, 1993).

Biochar is a charcoal-like material derived from biomass pyrolysis under low oxygen environments and relatively low temperatures (< 500°C) (Chun et al., 2004; Lehmann, 2007). As a result, the biomass is not fully carbonized and the relative carbonized vs. non-carbonized fraction determines its sorption character (Chen et al., 2008); the greater is this ratio, the higher will be the potential sorption ability of the material. Physical and chemical properties of biomass will vary as a function of the source biomass and pyrolysis conditions (Zhang et al., 2015). Because of the strong affinity of atrazine for organic functional groups, many have proposed the use of biochar as a sorbent in agricultural soils for prevention of atrazine losses to surface and groundwaters (Loganathan et al., 2009; Zhao et al., 2013; Mandal et al., 2017). Indeed several studies have concluded that on an equal mass basis, biochar often sorbs more atrazine than activated carbon and soil organic matter (Cao et al., 2009; Zhao et al., 2013; Deng et al., 2014). Further, Gonzalez et al. (2016a,b) suggested the use of biochar as a pesticide filtration media in “blind inlets,” structures designed to filter water and sediment in agricultural surface runoff around field depressions before drainage into a tile drain. In a 6-year study that utilized limestone and soil as a filtration media in a blind inlet, Gonzalez et al. (2016b) showed that atrazine transport from U.S. Midwestern fields was reduced by 57%. Several types of non-point drainage water treatment structures have been utilized for removing pollutants from flowing water (Penn et al., 2017); however, none have ever been constructed with biochar as a filtration media, as suggested by Gonzalez et al. (2016a).

Optimization of the design of a pollution-control structure for removing atrazine requires quantification and understanding of the strength and kinetics of sorption reactions. Batch isotherms is the traditional technique for this purpose; however, two approaches that may provide additional insight are isothermal titration calorimetry (ITC) and flow-through sorption experiments. The ITC provides real-time data as reactions proceed, thereby providing information about kinetics, energy of sorption, and in some cases, changes in reaction mechanisms. Thus, ITC precisely and directly measures the heat of reaction in real-time as a sample is titrated with known solutions. The degree and timing of the change in heat, either exothermic or endothermic, can serve as an indicator of the extent to which the reaction has taken place. In combination with benchtop experiments, the ITC has been used to (a) assess kinetics of ammonium exchange in zeolites (Penn et al., 2010) and phosphorus sorption onto soils and minerals (Lyngsie et al., 2014; Penn et al., 2014), (b) elucidate mechanisms of sorption (Penn and Warren, 2009; Lyngsie et al., 2014), (c) investigate dissolution of solids (Kallay et al., 1997), solution complexation reactions (Wu et al., 1997), and (d) to directly measure thermodynamic constants (Ohyama and Cowan, 1995; Martin et al., 1997). The ITC has the distinct advantage of directly measuring the heat of reaction; thermodynamic constants such as enthalpy, entropy, and Gibbs free energy are typically only indirectly estimated by conducting sorption isotherms at several different temperatures and applying the results to the Van Hoff equation.

Another useful tool within the realm of design of non-point pollution-control structures is flow-through sorption experiments, which simulates the use of filter media in its intended use through the constant addition of reactants, removal of reaction products, and a limited contact time. Depending on the pollutant, flow-through analysis will provide values of sorption that are different from traditional batch isotherms (Davidson et al., 1968; Kookana et al., 1992; Stoner et al., 2012). The results from flow-through studies can be directly used in the design of a drainage water filtration system, for example, a phosphorus removal structure (Penn et al., 2016).

The objective of this study was to investigate the kinetics and thermodynamics of atrazine sorption to biochar using two different approaches: ITC and flow-through sorption cells. Knowledge gained will provide valuable information for future design of pollution-control structures intended to filter atrazine from non-point drainage.

## Materials and methods

### Biochar characterization and atrazine preparation

Biochar was derived from pyrolysis of oak (Quercus spp.) at 425°C. The material was sieved to produce biochar particles from 2 to 5 mm in diameter. General characterization included pH, measured with an ion-specific electrode (1:5 solid:solution ratio, equilibration time of 30 min), total carbon and nitrogen by dry combustion (LECO, St. Joseph, MI), and surface area by Brunauer-Emmett-Teller (BET) nitrogen adsorption TriStar II surface area analyzer (Micromeritics Instrument Corp., Norcross, GA, USA) with a degassing temperature of 300°C for 3 h. This analysis was performed by the Materials Engineering Department at Purdue University (West Lafayette, IN, USA).

Atrazine solutions were prepared from 98.8% purity Pestanal® analytical grade atrazine (Fluka, St. Loius, MO, USA). The same source was used for all experiments.

### Isothermal titration calorimetry (ITC) experiments and accompanying atrazine sorption

All ITC experiments were conducted on a TAM IV ITC (TA Instruments, Newark, DE) at 25°C. The ITC had a “short term noise” level < 0.01 nJ s^−1^, baseline drift < 40 nJ s^−1^, accuracy < 2%, and precision ±100 nJ s^−1^. Heat rate measurements were recorded every 4 s. Two types of titration experiments were conducted on the biochar sample using three different atrazine solution concentrations for each experiment, in a matrix of 0.01 M CaCl_2_: 0.01, 3, and 20 mg atrazine L^−1^. For the first experiment, 25 mg of biochar suspended in 0.8 mL DI water was titrated 25 times with 0.01 mL atrazine per titration every 10 min. The biochar-water suspension contained in a Hastelloy vessel was constantly stirred during the experiment. Under the same conditions, the biochar was also titrated with a single addition of 0.25 mL atrazine solution, equal to the sum of the 25 titrations in the previous experiment. The resulting heat response was monitored for 5 h after the titrations. Blanks were measured by titrating the atrazine solutions into 0.8 mL of DI water, with no biochar, employing the same conditions as before. All experiments were duplicated and blanks were subtracted from the biochar sample measurements. The pH was monitored during the experiments with a pH micro-probe. In addition to the blanks, DI water was titrated into the 20 mg L^−1^ atrazine solution, and atrazine solution was titrated into a solution of the same atrazine concentration to explore the heat of atrazine dilution since the blanks (i.e., atrazine titrated into water) produced very high heat rates for the two highest atrazine concentrations.

To further explore the thermodynamics of atrazine sorption onto the biochar, an accompanying benchtop isotherm sorption kinetic experiment was conducted to simulate the ITC experiments. Using the same solid:solution ratio employed in the ITC experiment, biochar samples were treated with atrazine solution volumes equivalent to the 25 titrations and equilibrated in 30-mL Teflon tubes for the same time period i.e., 10 min intervals, at 25°C. Duplicated samples (and blanks containing no biochar) were lightly shaken on a reciprocating shaker during that time until removal and subsequent filtration through a 25-mm PTFE filter. Filtered samples were analyzed for atrazine and its metabolites 2-hydroxy atrazine (2HA), desisopropyl atrazine (DIA), and desethyl atrazine (DEA).

### Flow-through sorption experiments

A flow-through sorption experiment was conducted to investigate atrazine sorption to biochar under conditions similar to a pollution control structure, and measure the impact of contact time. The flow-through experiments were completed using a setup previously described in detail by Penn and Bowen (2017). Briefly, 3.5 g biochar was mixed with 1.5 g of lab grade sand (pure silica sand, 14808-60-7; Acros organics, Morris Plains, NJ, USA) to meet the desired pore volume of 3.15 mL, and placed in a 47-mm diameter flow cell containing a 0.45 μm filter. A 1 mg L^−1^ atrazine solution was continuously applied to the flow cell with a Mariotte bottle, with the bottom of the flow cell connected to a peristaltic pump that was adjusted to specified flow rates. The flow-through experiment was conducted at three different flow rates to achieve three desired contact times, where contact time is calculated as sample total pore volume divided by flow rate. The flow rates used were 4.2, 0.63, and 0.32 mL min^−1^ to achieve a contact time of 45, 300, and 600 s, which are common for field scale pollution-control structures (Penn et al., 2017). Each flow-through cell was duplicated.

All outflow was collected and separated every 10, 90, and 180 min for the 45, 300, and 600 s contact times, respectively. All samples were weighed to determine the volume that flowed through the biochar at each sampling. To achieve similar atrazine loads (i.e., mass atrazine added per mass of biochar), the experiments designed to achieve a contact time of 45, 300, and 600 s were conducted for 3, 24, and 48 h. A blank containing 12.4 g of pure silica sand with no biochar was included. All outflow and inflow samples were analyzed for atrazine and metabolites.

### Analysis of atrazine and metabolites

Samples were analyzed for atrazine and metabolites using a Waters Acquity ultra-pressure liquid chromatography (UPLC) system with an autosampler and coupled with mass detection and MassLynx v.4.1 chromatography manager software (Milford, MA). The mobile phase consisted of Optima grade solvents (Thermo Fisher Scientific Inc., Waltham, MA, USA): (A) 0.2% formic acid in 40% methanol and (B) 0.2% formic acid in 40% methanol +60% acetonitrile. Separation of atrazine and metabolites was performed at 45°C on a 100 × 2.1 mm i.d., 1.7 μm Waters Acquity UPLC BEC C18 analytical column with a 2.1 × 5 mm Acquity UPLC BEH C18 1.7 um Vanguard pre-column (Waters Corp., Milford, MA) with a flow rate of 0.30 mL min^−1^ using the following gradient: 0–1.5 min, 55% A; 1.5–2.0 min, 25% A; 2.0–3.0 min, 0% A; 3.0–4.0 min, 55% A. The autosampler was kept a 10°C to minimize atrazine degradation.

Detection and confirmation were performed using a Waters Acquity TQD (Waters Corp., Milford, MA) tandem quadrupole mass (MS-MS) detector in the Multiple Reaction Monitoring mode. The conditions of the MS-MS detector were as follow: voltages in capillary, cone, extractor, and RF lens were 0.61, 20, 3.0, and 0.1 kV, respectively; temperature of the source and desolvation were 150 and 400°C, respectively; and the gas flow of the desolvation and cone were 850 and 20 L h^−1^, respectively. Quantification was performed using the parent and daughter ions for each compound, atrazine: 216.06 and 174.00, 2-HA 198.00 and 156.00, DIA 173.99 and 132.03, and DEA 188.02 and 146.01, respectively. Five-point calibration curves (0.02–60 μg L^−1^) for all compounds were generated by using external standards prepared from certified solutions (Chem Services, West Chester, PA, USA). A 50-μL injection volume was used for both standards and samples. The retention times were 0.79, 0.97, 1.25, and 2.94 min for 2-HA, DIA, DEA, and atrazine, respectively. Limit of detection (LOD) for atrazine, 2HA, DIA, and DEA are 0.0072, 0.014, 0.24, and 0.0057 μg L^−1^, respectively. These LOD's were calculated following USEPA (2010).

For all experiments, no atrazine metabolites and/or only traces were detected, thus, only outflow and inflow atrazine concentrations were used to calculate discrete removal (%) at each sampling time. Atrazine loading and cumulative removal was calculated using the inflow and outflow atrazine concentration, flow rate, and mass of biochar. All values were averaged over replication. The relationship between cumulative atrazine added to biochar and cumulative atrazine removal was tested among the three contact times to determine whether the relationships (slope and intercept) were significantly different from each other at *p* = 0.05. The null hypothesis was that one equation could be used to describe cumulative atrazine addition vs. cumulative atrazine removal for all three contact times. This was tested by using a series of “contrast” statements in SAS (Sas and Guide, 2003) to determine whether the slope and intercept were significantly different based on contact time.

## Results and discussion

### Thermodynamics of atrazine dilution

**Figure 1:**
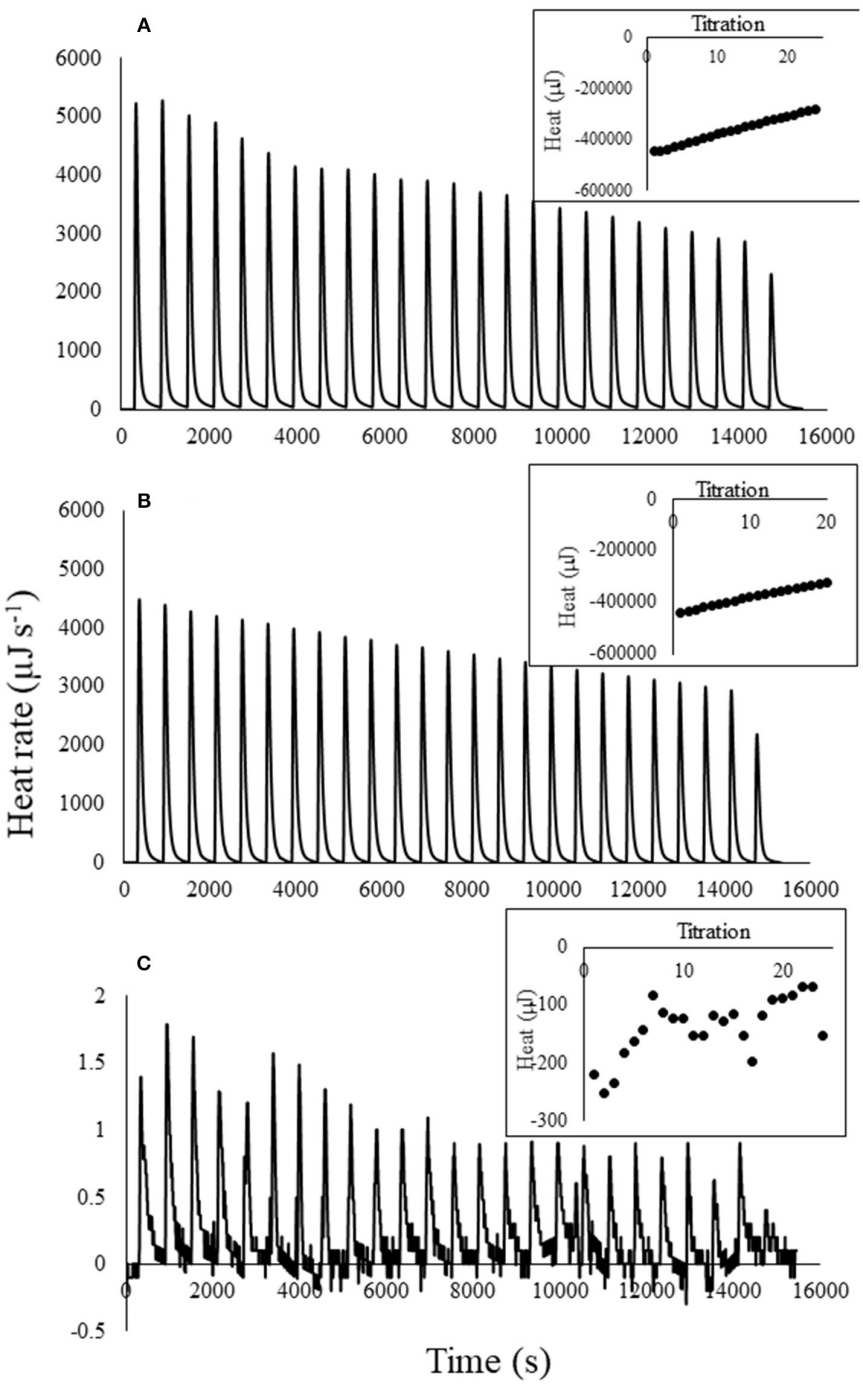
Thermograms for 25 titrations (0.01 mL per titration) of **(A)** 20 mg L^−1^ atrazine solution into water, **(B)** water into 20 mg L^−1^ atrazine solution, and **(C)** 20 mg L^−1^ atrazine solution into 20 mg L^−1^ atrazine solution. Inset illustrates the heat curves produced by integration of the thermogram peaks for each of the 25 titrations.

Titration of atrazine into water, which served as the blank for atrazine titrations into biochar, was highly exothermic for the highest atrazine concentration (Figure 1A). Heat of titration was proportional to atrazine concentration. Figure 1A shows the thermogram for titration of 20 mg L^−1^ atrazine into water; titration of the 3 and 0.01 mg L^−1^ atrazine solutions displayed less energy than the 20 mg L^−1^ solution. The 0.01 mg L^−1^ atrazine titration into water released the least heat, with values similar to water titrated into water (data not shown). This exothermic heat is not due to the protonation or de-protonation of the atrazine since the pKa of atrazine is only 1.68 (Wang et al., 1992) and the pH of the atrazine solution was 5.89, which was the same as the DI water used in all experiments. For comparison, water titration into the 20 mg L^−1^ atrazine solution produced a similar thermogram as atrazine titrated into water (Figure 1B), while titration of atrazine into atrazine produced very little heat (Figure 1C). This suggests that simple dilution of atrazine produces considerable heat. For example, application of the measured heat of dilution to a real-world scenario of mixing atrazine into a 3,840 L spray tank commonly used on farms would result in 7.9 kJ of heat, enough to raise the temperature of the water in the tank 0.5°C.

Position number five on the atrazine ring contains a lone set of electrons and is therefore hydrophilic (Laird and Koskinen, 2008). However, since the atrazine was already solvated before titration into water, it is unknown what reactions are taking place upon dilution that resulted in the strong exotherms. One possibility is that the alkyl side chains of atrazine can disrupt the H-bonding network of water (Laird and Koskinen, 2008). Using a similar calorimetric technique, Nunes et al. (2011) also found that titration of atrazine into water was highly exothermic.

### Thermodynamics of atrazine sorption to biochar

Basic biochar characterization revealed that the sample was typical compared to previous studies (Chun et al., 2004; Zhang et al., 2013; Deng et al., 2017). Total surface area was 332.86 m^2^ g^−1^, and pH was 8.98. Being a carbon-based material, total carbon comprised much of the media with 83.3% carbon and 0.3% nitrogen (by weight). Being derived from oak, nitrogen content was relatively low compared to biochar derived from agricultural materials that are often rich in nitrogen, such as manure or certain plant parts. However, nitrogen content was similar to biochar derived from another nitrogen-deficient material; corn straw (Zhao et al., 2013).

**Figure 2:**
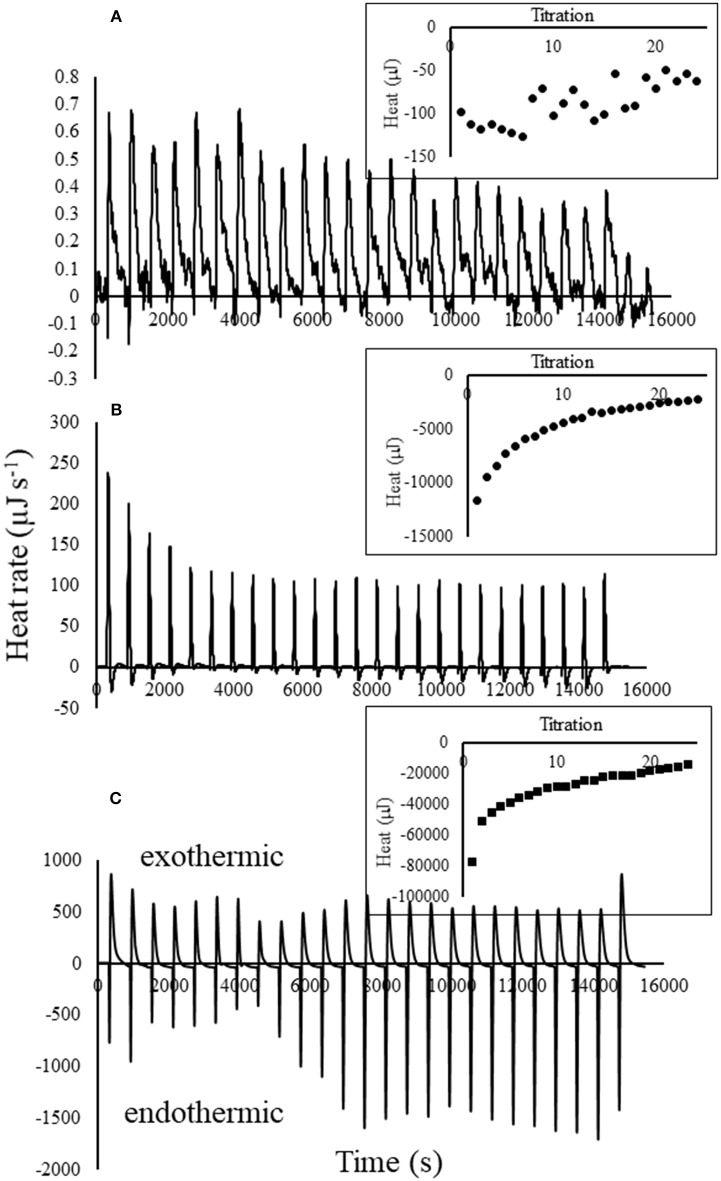
Thermograms with blanks subtracted for 25 titrations of **(A)** 0.01, **(B)** 3, and **(C)** 20 mg L^−1^ atrazine solution into 25 mg of biochar in 0.8 mLs water. Inset illustrates the heat curves produced by integration of the thermogram peaks for each of the 25 titrations.

Titration of atrazine into biochar, after subtracting the blanks, was exothermic for all atrazine concentrations (Figure 2). Clearly, higher atrazine concentrations resulted in greater exotherms; heat produced from the 20 mg L^−1^ atrazine solution was approximately eight times more than the 3 mg L^−1^ solution, which produced 100 times more heat than the 0.01 mg L^−1^ solution. Similar to our results, Nunes et al. (2011) found that atrazine (10,000 mg L^−1^ in methanol) sorption onto magadiite was highly exothermic. In contrast, Deng et al. (2014), found that sorption of atrazine (0.5–20 mg L^−1^) to cassava (*Manihot esculenta*) derived-biochar was weakly endothermic. However, that study did not directly measure the heat from reactions; instead, the enthalpy was estimated from a sorption isotherm conducted at several different temperatures. It is also important to note that there is great variability between different biochar materials as a function of carbon source and pyrolysis parameters. It is important to note that the strong heat of reaction exhibited by the 3 and 20 mg L^−1^ atrazine solutions were not due to pH changes. Titration of 0.01 *M* HCl into biochar initially reduced the pH 0.4 units at each titration, followed by rebounding back to near the original level. This pH-buffering exhibited by the biochar resulted in exothermic heat of only 9 μJ s^−1^, compared to the nearly 500 μJ s^−1^ produced from titration of 20 mg L^−1^ atrazine into biochar (Figure 2), making the heat from small pH changes, negligible. For the lowest atrazine concentration tested, titration into biochar only decreased the pH 0.028 units per titration.

While the net heat resulting from atrazine titrations into biochar were exothermic as evident from integration of each titration peak shown in the insets, there were some endotherms occurring as well. For example, the thermograms in Figure 2 indicate that an exotherm occurred immediately after each titration of atrazine into biochar, followed by a smaller endothermic reaction. Keep in mind that heat measured by the calorimeter is the result of multiple reactions that are taking place, some of which are simultaneously occurring.

Another similarity among the thermograms and heat curves for each atrazine solution shown in Figure 2 is that the initial titrations display the strongest exotherms and weakest endotherms, followed by the exotherms and endotherms becoming progressively weaker and stronger, respectively, indicating a changing magnitude in the degree of the reactions responsible for them. Using titration calorimetry, other studies have also shown a decreasing heat response with further additions of titrant into soils, minerals, and by-products (Penn and Warren, 2009; Penn and Zhang, 2010; Nunes et al., 2011; Penn et al., 2014; Almeida et al., 2018). This is expected as the reactants (i.e., biochar sorption sites) are further consumed and converted into reaction products with each additional titration.

**Figure 3:**
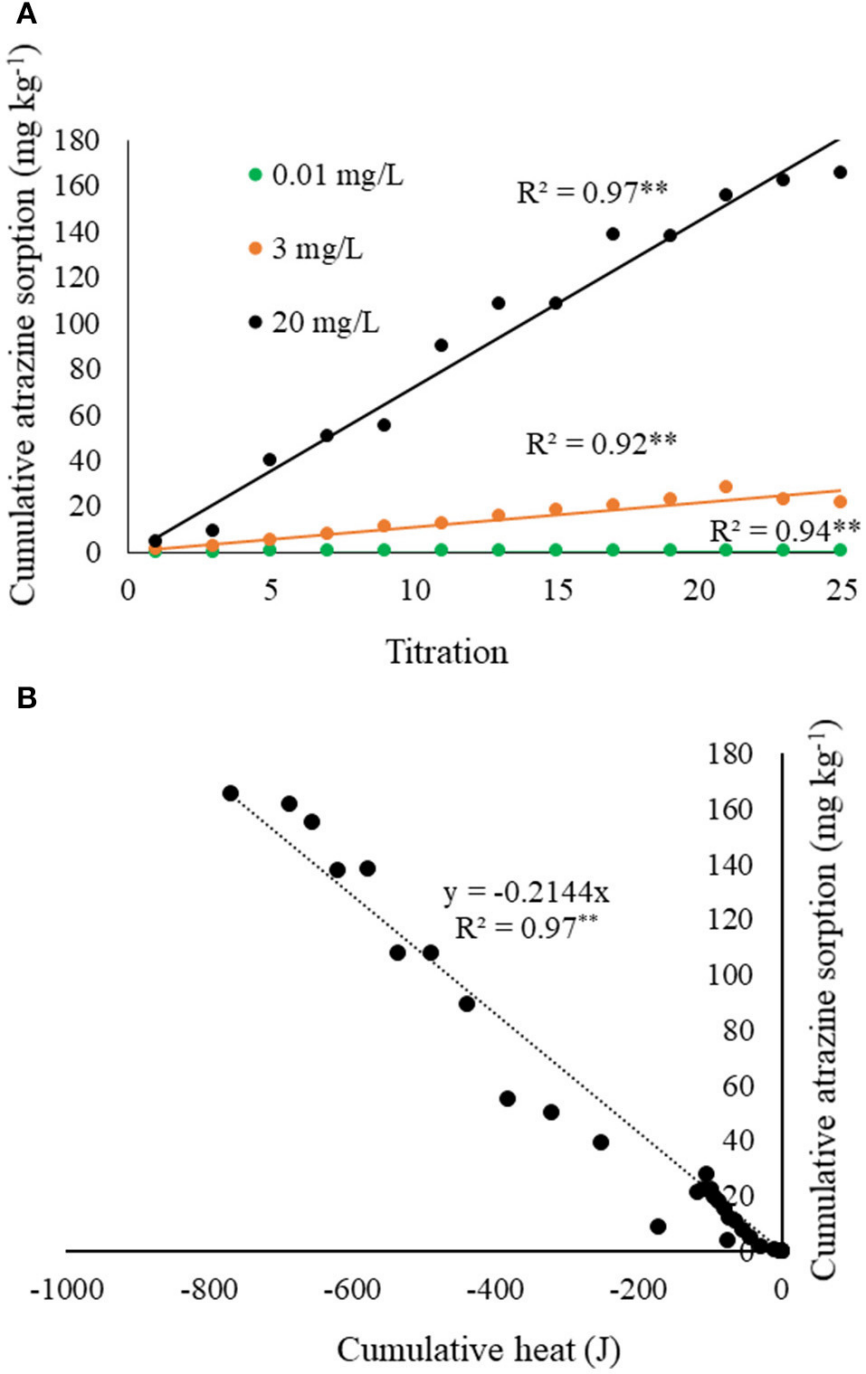
**(A)** Measured cumulative atrazine sorption as a function of titration number for each atrazine concentration tested, corresponding to the ITC experiment shown in Figure 2. **(B)** Cumulative atrazine sorption from **(A)** as a function of cumulative heat from each of the corresponding titrations shown in Figure 2.

The complimentary atrazine sorption isotherms (Figure 3A), which simulated the calorimeter titration experiments, provided further insight into the thermograms and heat curves. First, every titration of atrazine into biochar, regardless of atrazine concentration, resulted in nearly 100% sorption. As a result, Figure 3 clearly shows linear atrazine sorption over the range of the 25 titrations. Together with Figure 2, results in Figure 3 indicate that the exotherms are at least partly a result of atrazine sorption to biochar, with exothermic heat production being proportional to atrazine sorption. Combining the heat and sorption data, a plot of cumulative heat against the corresponding cumulative atrazine sorption produces a linear relationship (Figure 3B). From the slope of this relationship, the net enthalpy of atrazine sorption to this biochar sample is calculated to be −4,231 ± 130 kJ mole^−1^. Note that this is considered a “net” sorption enthalpy because the heat measured from the titration of atrazine into biochar likely involves multiple sorption reactions. The degree of sorption was constant for every titration within each experiment (Figure 3), yet the heat of reaction for each titration decreased with each titration (Figure 2), suggesting a change of sorption mechanism and/or other accompanying reactions are shifting in type or magnitude. This is expected as several mechanisms for atrazine sorption onto soil minerals and organic functional groups have been shown, such as physical fixation, ionic bonding, covalent bonding, hydrogen bonding, van der Waals forces, and hydrophobic interactions (Weber, 1993; Welhouse and Bleam, 1993; Barriuso et al., 1994; Ma and Selim, 1996). Laird and Koskinen (2008) noted that two or more mechanisms often contribute to the interaction energy between atrazine and organic functional groups.

The observation of both exotherms and endotherms also indicates that several sorption mechanisms were occurring with titration of atrazine into biochar. In a review of the literature, Laird and Koskinen (2008) demonstrated the complexity of atrazine sorption in that multiple studies showed that as temperature increased, atrazine sorption decreased (i.e., exothermic), increased (i.e., endothermic), or remained the same. Zhao et al. (2013) also concluded that atrazine sorption onto biochar derived from corn straw involved both chemical bonding and physical sorption, and depending on sample pre-treatment, was exothermic or endothermic. In addition to the several sorption moieties in the atrazine molecule, other possible explanation for the diversity in mechanisms is the presence of both carbonized and non-carbonized fractions in biochar. Specifically, sorption of an organic molecule by a carbonized fraction is characterized by nonlinear Langmuir isotherms, while the non-carbonized fraction sorbs by a linear partitioning mechanism (Chiou and Kile, 1998; Accardi-Dey and Gschwend, 2002).

Since the pH of the biochar-atrazine solution was above neutral (8.23–8.98), it is unlikely that atrazine sorbed to biochar by cation exchange, as atrazine exists in an uncharged state at non-acid pH levels (Weber, 1993). Previous studies have speculated on the mechanisms of atrazine sorption to biochar. Zhang et al. (2015) noted that atrazine sorption onto a sludge-derived biochar was mostly due to H-bonding. Using Fourier transform infrared spectroscopy, Deng et al. (2014) concluded that physical partitioning via H-bonding with carboxyl groups was the main sorption mechanism because the calculated enthalpy was < 40 kJ mole^−1^. Cao et al. (2009) also concluded a partitioning mechanism for atrazine sorption onto biochar, because their biochars produced from dairy manure were rich in organic carbon, and the observation of a linear isotherm. According to Zhang et al. (1990), linear sorption of an organic solute is due to physical sorption, not formation of a surface chemical bond. The authors further illustrated that linear partitioning is indicated by heat sorption (i.e., endotherm), while formation of a chemical bond between the solute and the surface releases heat (i.e., exotherm).

### Kinetics of atrazine reactions with biochar

**Figure 4:**
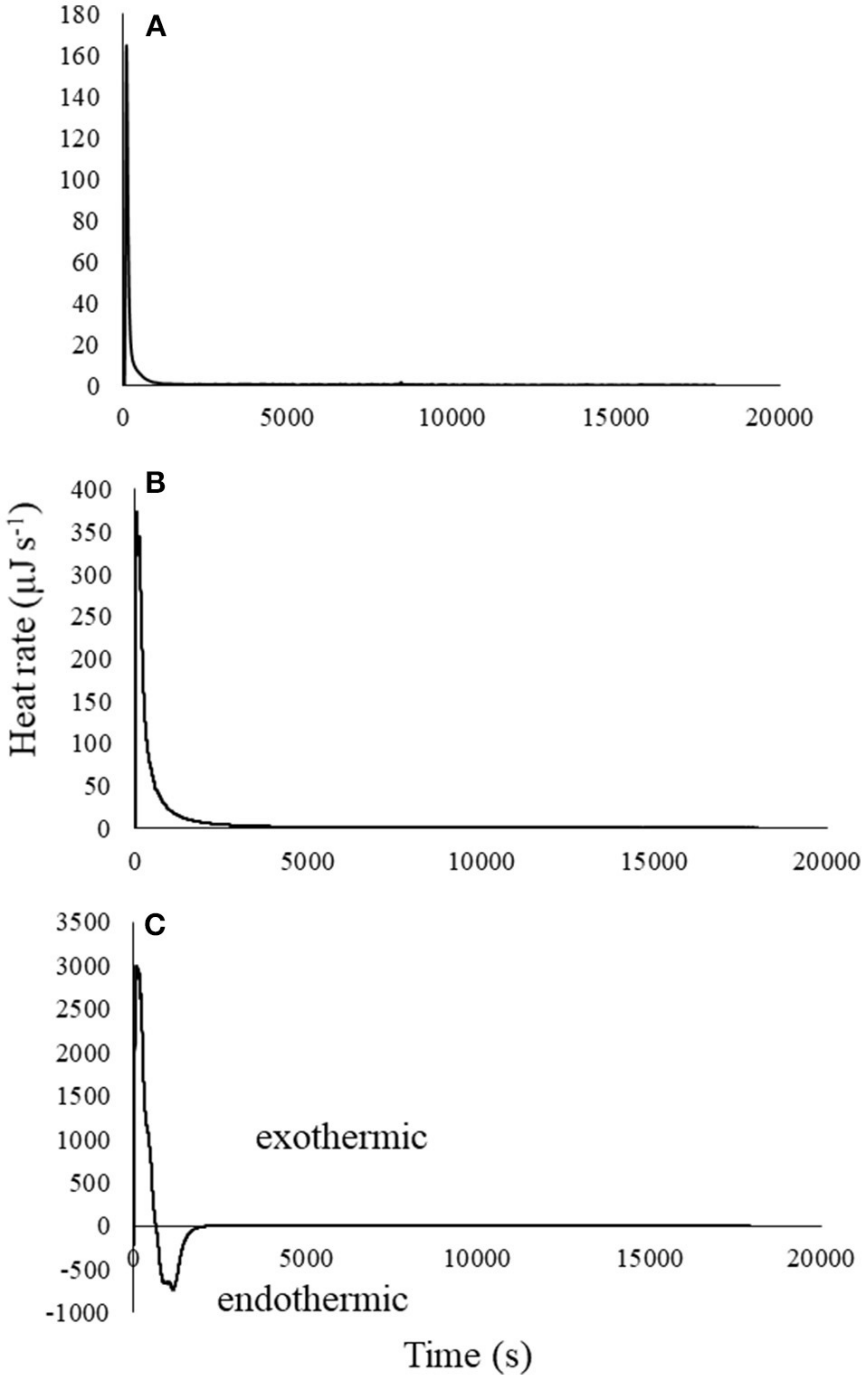
Thermograms with blanks subtracted for single titration (0.25 mLs) of **(A)** 0.01, **(B)** 3, and **(C)** 20 mg L^−1^ atrazine solution into 25 mg of biochar in 0.8 mLs water.

Figure 4 shows thermograms for a single titration of atrazine into biochar for each of the three atrazine concentrations. Similar to Figure 2, the heat of reaction was proportional to the concentration of atrazine titrated, and therefore to the degree of atrazine sorption. This single titration technique permits examination of the kinetics of atrazine-biochar interactions. Although the highest atrazine concentration displayed both an exotherm and endotherm (Figure 4A), the net reaction was exothermic, with the endotherm dominating after the exotherm reached its peak, similar to the 25-titration experiment previously discussed. Again, this supports the notion that multiple sorption reactions are taking place. Similar to Figure 2, endotherms could also be occurring in Figure 4 for the two lower atrazine concentrations (Figures 4A,B), although they may be masked by the greater exotherm that overlaps with the endothermic reaction. Recall that Figure 2 also displayed both an exotherm and endotherm for most titrations (similar to Figure 4A), indicating that the reactions overlapped some as the exotherm initially dominated and then decreased with each additional titration, while endotherms increased.

**Figure 5:**
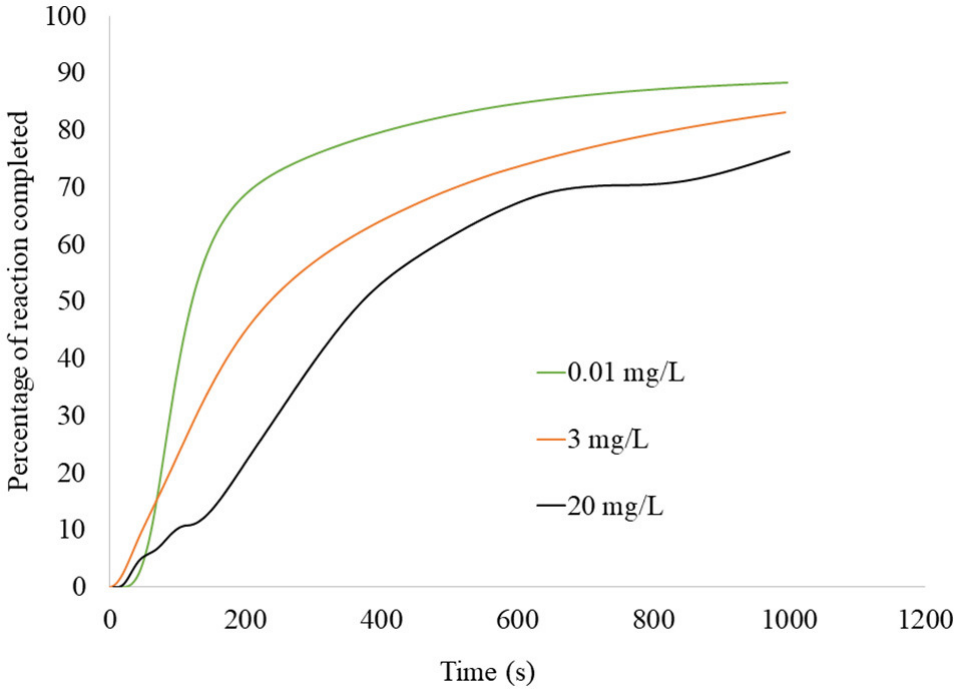
Percentage of atrazine reaction onto biochar as a function of time, for the single-titration calorimetry experiment shown in Figure 4 conducted using three different atrazine concentrations (0.01, 3, and 20 mg L^−1^). Curves produced by integration of the thermograms in Figure 4, followed by expressing the cumulative heat measured at each time as a percentage of the total cumulative heat.

Since the accompanying benchtop test indicated 100% sorption (Figure 3), it can be assumed that net heat of reaction indicates the degree of sorption, relative to the total amount that was sorbed for each atrazine concentration tested. Figure 5 was developed through integration of the peak in order to illustrate the fraction of equilibrium completed with time. Figure 5 indicates that atrazine-biochar reactions occurred in at least two steps with an initial fast reaction followed by a general slowing down with time. Several studies have also noted the existence of an initial fast reaction of atrazine with biochar, followed by a slower step (Deng et al., 2014; Zhang et al., 2015; Mandal et al., 2017). If the exotherms and endotherms indicate surface chemical bond formation and physical partitioning, respectively (as previously discussed), then surface chemical bonding occurred much faster compared to the later partitioning, which is expected. Again, consider that the heat of reaction measured by the calorimeter is an overall net reaction indicator, not just of the chemical sorption of atrazine. Deng et al. (2017) observed that biochar derived from cassava waste, removed atrazine slowly and through endothermic physical partitioning. There are likely multiple reactions taking place, as well as mass transfer/physical phenomena that are occurring simultaneously (Sparks, 2003). This would also partly explain the observation of the two-stage sorption. For example, Leenheer and Ahlrichs (1971) found that sorption of carbaryl and parathion onto soil organic matter occurred in two different stages, with the latter stage being rate limiting as the solute slowly diffused into the interior of the adsorbent. Indeed, this is possible for our biochar sample since the sorption of aromatic hydrocarbons to a wood char sample has been shown to occur through both π-electron interactions and a slower pore-filling mechanism (Zhu et al., 2005; Nguyen et al., 2007; Wang and Xing, 2007).

At 400 s, at least 50% of the equilibrium was completed for all atrazine concentrations. Deng et al. (2014) also showed rapid sorption of atrazine onto a biochar-amended soil sample with 50% occurring in < 35 min. The high degree (Figure 3), energy (Figure 2), and rate (Figure 5) of atrazine sorption onto biochar suggests that this biochar sample is an excellent candidate for use in passive filtration of agricultural drainage water that is rich in atrazine. Relative to the cumulative sorption (as indicated by heat) for each experiment, initial atrazine concentration was inversely proportional to the time required for achieving equilibrium. That is, the 0.01 mg L^−1^ atrazine concentration achieved any given degree of reaction completion more rapidly than the higher concentrations (Figure 5). In general, to reach equilibrium, higher concentrations would require a greater extent of sorption compared to lower concentrations, and therefore would require more time. For example, to achieve 50% completion of reaction, Figure 5 indicates that it would require ~120, 250, and 380 s for 0.01, 3, and 20 mg L^−1^ atrazine solutions, respectively.

### Filtration of atrazine with biochar under flowing conditions

**Figure 6:**
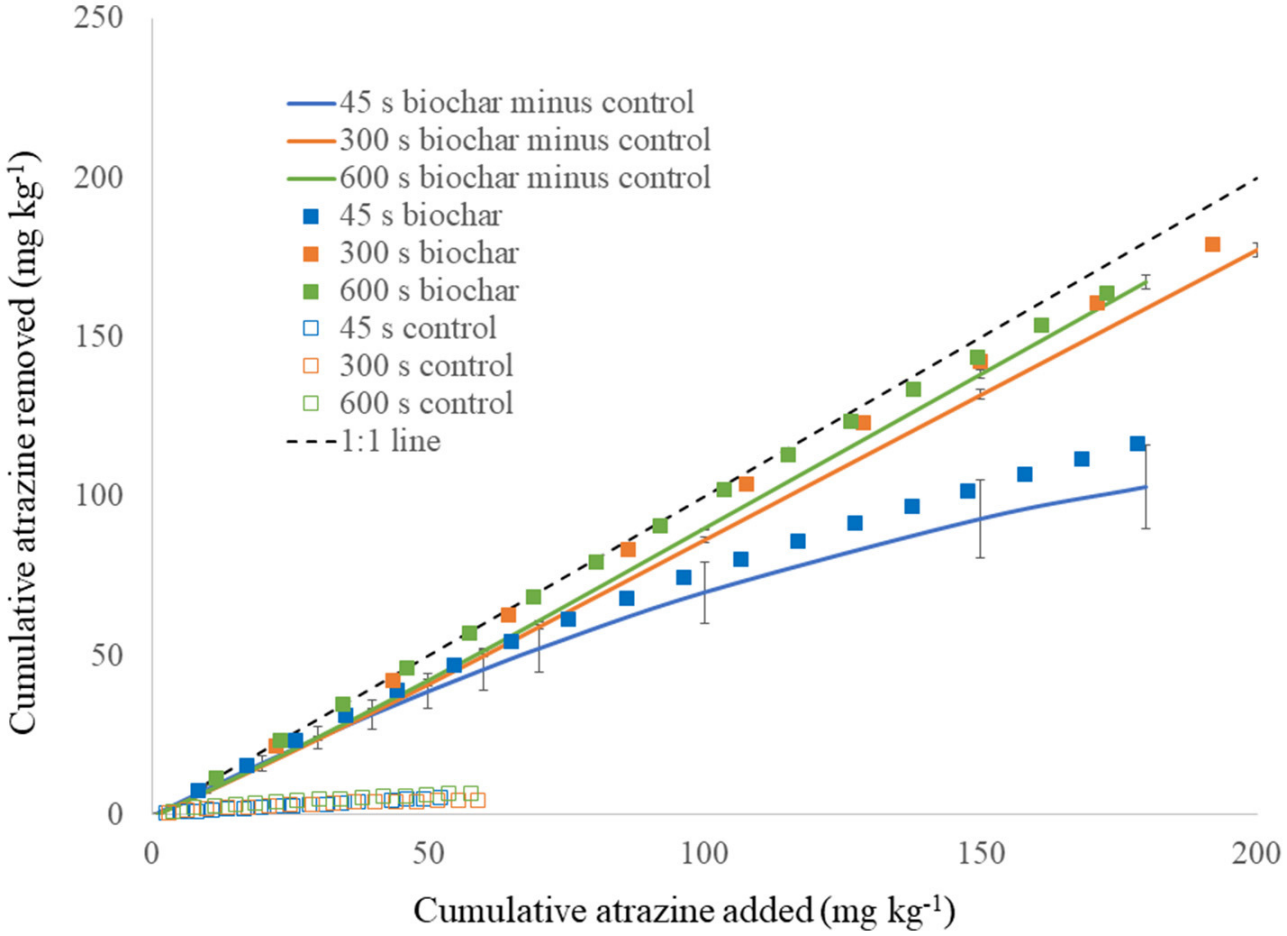
Atrazine (1 mg L^−1^) removal under flow-through conditions by biochar and pure silica sand (control) at three different contact times (45, 300, and 600 s), shown at a function of cumulative atrazine loading. Solid lines indicate biochar minus control. 1:1 line illustrates 100% removal for comparison. Vertical bars are standard deviation.

Contact time had a profound impact on atrazine sorption to biochar under flow-through conditions (Figure 6). Figure 6 shows the cumulative amount of atrazine removed for each contact time, after normalization for atrazine loading to the media. The control (sand only) removed a small amount of atrazine at maximum of 6.8 mg kg^−1^. Regardless, the control was subtracted from the biochar, which contained sand mixed into it for achieving the desired pore volume. Contact times of 300 and 600 s produced similar results, although they had statistically different slope values (*p* < 0.01) with slightly greater sorption at 600 than 300 s. However, atrazine sorption was dramatically less for the 45 s contact time compared to the 300 and 600 s contact time (both slope and intercept were statistically different for 45 s compared to 300 and 600 s). As suggested from the single-titration thermograms and their respective integrations that indicated the degree of reaction completion with time (Figure 5), increasing contact time clearly increased atrazine sorption to biochar (Figure 6). According to Figure 5, at 45 s a much smaller percentage of the sorption reaction is completed compared to 300 and 600 s. Notice also that due to the decreasing slope in Figure 5, atrazine sorption does not increase nearly as much from 300 to 600 s compared to 45–300 s. This is reflected in the flow-through results of Figure 6 with an appreciable increase in atrazine sorption when increasing the contact time from 45 to 300 s, but much less increase from 300 to 600 s.

Based on the shape of the cumulative flow-through curve in Figure 6, estimates can be made for sizing pollution-control structures that would utilize the biochar used in this study (Penn and Bowen, 2017). For this example, we will assume a typical Midwestern U.S. tile drain outlet that produces 7.68 million L of drainage per year, from a contributing area of 8–16 ha and having the same concentration of atrazine utilized in the flow-through experiment. If the atrazine treatment goal was to remove 50% of the annual load, then it would require ~32 Mg of biochar if the pollution-control structure was designed to operate with a 45 s contact time, compared to only 4 Mg biochar for a 300 s contact time. Clearly, there is an economic advantage to designing the hypothetical structure for operating at 300 s contact time.

## Conclusions and implications

Multi- and single-titrations of oak-derived biochar with atrazine was net exothermic, with the magnitude of the exotherm being proportional to the amount of atrazine sorption (Figures 2, 4). Although each titration was net exothermic, thermograms clearly indicated an initial exotherm followed by an endotherm, suggesting at least two different sorption mechanisms had occurred. Single-titration experiments likewise showed that the initial exotherm occurred fast compared to the slower endotherm that followed (Figures 4, 5). This was not surprising since some studies have reported atrazine sorption as exothermic, while others reported endothermic (Laird and Koskinen, 2008). The presence of distinct strong exotherms and weaker endotherms combined with observation of an initial fast sorption phase followed by a slower phase suggests that the biochar sample may have sorbed atrazine initially through formation of chemical bonds followed by a slower physical partitioning mechanism, perhaps H-bonding as shown in previous studies (Zhu et al., 2005; Nguyen et al., 2007; Wang and Xing, 2007). The slower and weaker (i.e., less heat) secondary phase could also be due to a physical limitation with atrazine slowly diffusing into the pores of biochar.

The observation that exotherms were stronger with initial titrations and became weaker with each progressive titration, even though the measured atrazine sorption was 100%, suggests that the initially sorbed atrazine was held stronger than atrazine added with subsequent titrations. The net of all atrazine sorption reactions, regardless of reaction mechanism, resulted in an enthalpy of −4,231 ± 130 kJ mole^−1^ sorbed atrazine, suggesting that atrazine was held onto this biochar sample with great strength. The strength of atrazine sorption can have implications on its environmental fate. For example, while it is desirable for atrazine to be held tightly onto biochar, such bonding could diminish its capacity to be degraded by microorganisms; on the other hand, strong sorption prevents the transport of the chemical in moving water (Sparks, 2003). Gonzalez et al. (2016a) attempted to desorb atrazine from a biochar sample after it had been previously equilibrated with 0.05 and 0.1 mg L^−1^ atrazine solution. The authors found that shaking the sample with 0.01 M CaCl_2_ for 24 h released no atrazine.

The role of sorption kinetics onto the biochar sample was manifested in a practical manner through the flow-through sorption experiment. The flow-through sorption experiments represent conditions most representative of a pollution-control filter structure where new water and reactants (i.e., atrazine) are constantly flowing into it and the treated water is exiting the structure at the same rate. The existence of the dual-phase sorption observed in the thermograms and heat curves (Figures 2, 4, 5) had a clear impact on the effect of contact time in the flow-through tests. Specifically, with a rapid sorption phase occurring initially through ~50% of sorption occurring at 400 s followed by a slower phase (Figure 5), increase in contact time from 45 to 300 s dramatically increased atrazine sorption while an increase in contact time from 300 to 600 s minimally increased sorption (Figure 6). Contact time is a critical component in design of a pollution-control structure. Contact time can only be increased by decreasing the flow rate of water through the structure or increasing the total pore volume via increased mass of filter media in the structure; both options can have negative practical consequences. On the other hand, if increased contact time improves sorption, then less filter media would be required compared to a structure with a lesser contact time. However, decreasing the flow rate through a pollution-control structure reduces its capacity to treat non-point flow events, meaning that not all the event water would be treated. Increased mass of the sorption media obviously increases the total cost of the pollution-control structure. Therefore, pollution-control structures are most efficiently designed when the contact time is equal to the point where the initial fast reactions have been completed; increasing the contact time beyond that point would represent an unnecessary inefficiency in design (see Penn and Bowen, 2017, for details on efficient design of pollution control structures). Based on the flow-through data shown in this study (Figure 6), the most efficient contact time for this biochar sample at 1 mg L^−1^ atrazine inflow, is ~300 s.

While biochar can be used to filter atrazine under flowing conditions and contact times that are common to pollution-control structures, less is known about degradation of the compound after sorption. Unlike pollution-control structure built for removal of inorganics such as phosphorus (Penn et al., 2017), organic compounds such as atrazine can potentially biodegrade, possibly renewing the sorption sites. This would increase the lifetime of pollution-control structures intended to remove organic compounds such as atrazine. Future research is necessary to quantify this potential degradation for use in improving pollution-control structures using biochar to remove organic compounds.

## Author contributions

CP: conception, methodology, data management, statistics, original manuscript draft. JG: conception, methodology, editing manuscript. IC: methodology, execution, data management, editing manuscript.

### Conflict of interest statement

The authors declare that the research was conducted in the absence of any commercial or financial relationships that could be construed as a potential conflict of interest.
